# S100A8/A9 as a novel predictor for severe *Mycoplasma pneumoniae* pneumonia in children: A prospective observational study

**DOI:** 10.1016/j.clinsp.2026.101119

**Published:** 2026-09-07

**Authors:** Zihui Liu, Lihui Gao, Yun Cui, Yiping Zhou, Xi Xiong, Chunxia Wang, Yucai Zhang

**Affiliations:** a Department of Critical Care Medicine, Shanghai Children's Hospital, School of Medicine, Shanghai Jiao Tong University, Shanghai, China; b Core Facilities and Transformation Center, Institute of Pediatric Infection, Immunity, and Critical Care Medicine, Shanghai Children's Hospital, Shanghai Jiao Tong University School of Medicine, Shanghai, China; c Laboratory of Critical Care Translational Medicine, Institute of Pediatric Infection, Immunity, and Critical Care Medicine, Shanghai Children's Hospital, Shanghai Jiao Tong University School of Medicine, Shanghai, China; d Institute of Pediatric Critical Care, Shanghai Jiao Tong University, Shanghai, China

**Keywords:** Calprotectin S100A8/A9, Biomarker, Severe *Mycoplasma Pneumoniae* Pneumonia (SMPP), Child

## Abstract

**Objective:**

The calprotectin S100A8/A9 complex is released by stimulating neutrophils and monocytes, and inducing cytokine secretion, modulating the inflammatory response. The aim of present study was to investigate the role of S100A8/A9 in Severe *Mycoplasma Pneumoniae* Pneumonia (SMPP).

**Study design:**

We conducted a prospective study on pediatric *Mycoplasma Pneumoniae* Pneumonia (MPP) from December 2023 to May 2024. Patients were categorized into the SMPP group and non-SMPP (Non-*Mycoplasma Pneumoniae* Pneumonia, NSMPP) group based on hospitalization. Matched subgroups were formed according to age, sex, co-infection.

**Results:**

A total of 132 patients were included. Serum S100A8/A9 levels in SMPP were significantly higher than those in NSMPP group (5.877 [3.202, 9.257] vs. 4.349 [2.495, 6.902] μg/mL, *p* = 0.049). Both C-Reactive Protein (CRP) and Lactate Dehydrogenase (LDH) levels were significantly elevated in SMPP compared to NSMPP groups (*p* = 0.009 and *p* = 0.036, respectively). Multivariate *logistic* regression analysis revealed that S100A8/A9 levels is a key predictor for SMPP. The Area Under the ROC Curve (AUC) for S100A8/A9 levels in predicting SMPP was 0.601 (95% CI: 0.512‒0.685). Post-matching, the AUC increased to 0.714 (95% CI: 0.549‒0.845), with an optimal cutoff value of S100A8/A9 at 4.067 μg/mL, a sensitivity of 75%, and a specificity of 70%. Additionally, the AUCs for CRP and LDH were 0.701 and 0.690, respectively. The combined variables of S100A8/A9 and CRP achieved an AUC of 0.742 (95% CI: 0.580‒0.867), showing a relative advantage over S100A8/A9 alone.

**Conclusion:**

Our findings indicate that serum S100A8/A9 is a potential adjunct biomarker for predicting severe *Mycoplasma Pneumoniae* Pneumonia (SMPP) in children. The conclusion is preliminary exploration and requires further validation in larger, multicenter prospective cohorts.

## Highlights

• Elevated serum S100A8/A9 serves as a novel predictive biomarker for SMPP.
• S100A8/A9 is comparable to CRP and D-dimer for predicting SMPP progression.
• Early S100A8/A9 testing facilitates timely intervention to improve pediatric SMPP outcomes.
• Integrating S100A8/A9 into MPP risk stratification provides clinical benefit.

## Introduction

*Mycoplasma Pneumoniae* (MP) is a leading cause of community-acquired pneumonia among children.^1^ As an extracellular pathogen, MP colonizes respiratory tract epithelial cells and invades the cell membrane, triggering significant inflammatory responses and *Mycoplasma Pneumoniae Pneumonia* (MPP).^2^ Although most children with MPP recover fully, Severe MPP (SMPP) is characterized by more serious clinical symptoms and imaging findings.^3^ The rising incidence of SMPP has drawn increasing concern due to its diverse clinical manifestations and potential life-threatening extrapulmonary complications, such as myocarditis, pericarditis, hemolytic anemia, and thrombosis.^4^ Early identification of SMPP progression and prevention of fatal outcomes are critical clinical priorities.

Excessive or persistent inflammatory responses can lead to tissue damage and even organ dysfunction.^5^ In SMPP, an excessive inflammatory response is a key factor contributing to pulmonary injury and extrapulmonary organ dysfunction.^6^ To assess the risk of progression to SMPP, clinical indicators such as C-Reactive Protein (CRP), D-dimer, and Lactate Dehydrogenase (LDH) have been utilized as biomarkers for predicting SMPP.^7^, ^8^, ^9^ However, the low specificity of these markers limits their clinical utility. For instance, significantly elevated CRP levels have also been observed in patients with severe COVID-19 and are correlated with disease severity.^10^ D-dimer levels can be influenced by underlying comorbidities,^11^ and elevated serum LDH is commonly observed in various malignancies.^12^ Therefore, there is an urgent need to identify novel biomarkers with high specificity and sensitivity for MP infection.

Calprotectin, primarily a heterodimer of S100A8 and S100A9 (S100A8/A9), is stabilized in this form due to the relative instability of homodimers.^5^ It is constitutively expressed in neutrophils and monocytes, where it acts as a Ca^2+^ sensor and participates in cytoskeleton rearrangement and arachidonic acid metabolism.^13^ In MPP, leukocytes recruitment-particularly of neutrophils and monocytes-along with cytokines release, contributes significantly to lung injury and extrapulmonary complications.^14^ Bai S et al observed elevated S100A8/A9 levels in both Bronchoalveolar Lavage Fluid (BALF) and serum of children with MPP.^15^ However, serum S100A8/A9 levels were not compared between SMPP and Non-SMPP (NSMPP) cases in that study. Given that excessive S100A8/A9 can amplify inflammation by prompting macrophage cytokine release and worsening disease progression,^16^ we hypothesize that S100A8/A9 may serve as a potential predictor for SMPP. This study aims to investigate S100A8/A9 in pediatric MPP and explore its utility in the early diagnosis of SMPP.

## Materials and methods

### Study design and patient enrollment

Children diagnosed with MPP were admitted to the general ward or Pediatric Intensive Care Unit (PICU) at Shanghai Children’s Hospital between December 2023 and May 2024. The inclusion criteria were as follows: 1) Age ranging from 1-month to 18-years; 2) Patients who underwent chest radiography and MP testing, including serum MP-IgM antibody and real-time PCR for MP DNA in nasopharyngeal aspirates. Exclusion criteria included patients who had used antibiotics or immunosuppressive drugs, those with a hospital stay of less than 24-hours, those who had not signed the consent form, and those with concurrent underlying diseases including advanced tumor, genetic metabolic disorders, or autoimmune disease.

Patients were diagnosed with SMPP and Non-SMPP (NSMPP) during the hospitalization and were treated in accordance with the Guidelines for Diagnosis and Treatment of Mycoplasma Pneumonae Pneumonia in Children (2023 Edition).^17^ SMPP is defined as severe Mycoplasma pneumoniae pneumonia meeting the diagnostic criteria for severe Community-Acquired Pneumonia (CAP), as indicated by at least one of the following: 1) Prolonged fever: persistent high fever (≥39°C) for ≥5-days or fever lasting ≥7-days without evidence of defervescence, 2) Respiratory complications: presence of respiratory signs (e.g., wheezing, tachypnea, dyspnea, chest pain, or hemoptysis), often associated with complications such as plastic bronchitis, asthma exacerbation, pleural effusion, or pulmonary embolism, 3) Extrapulmonary involvement: presence of any extrapulmonary complications not yet meeting the criteria for critical illness, 4) Hypoxemia: resting Oxygen Saturation (SpO_2_) ≤93% on room air, 5) Radiologic criteria: dense consolidation involving ≥ 2/3 of a single lobe or involving two or more lobes (irrespective of the extent within each lobe), which may be accompanied by moderate-to-large pleural effusion or features of bronchiolitis, and diffuse infiltration in one lung, or infiltration involving ≥ 4/5 of both lungs with bronchiolitis signs, potentially combined with bronchitis and atelectasis due to mucus plugs, 6) Rapid progression: clinical deterioration with a > 50% increase in the radiographic lesion size within 24‒48 hours, 7) Laboratory markers: significant elevation in at least one of the following: C-Reactive Protein (CRP), Lactate Dehydrogenase (LDH), or D-dimer.^17^ The study protocol received approval from the local ethics committee and was conducted in compliance with the ethical standards stipulated in the Declaration of Helsinki (Ethics Committee of the Children’s Hospital affiliated to Shanghai Jiao Tong University, Approval number: 2023R099-F01). Informed consent was obtained from the patients’ parents or legal guardians. This study was conducted in accordance with the STROBE Statement.

### Observational variable

Demographic parameters, clinical manifestations and laboratory data were systematically collected using a pre-designed case report form during MPP, at the time of hospitalization and subsequent specified intervals. These parameters included age, sex, comorbidities, pathogens, extrapulmonary organs injury, and macrolide resistance. To assess lung injury, chest radiographs were independently reviewed by two experienced radiologists. The etiological diagnosis was inferred from epidemiological and clinical indicators. Diagnosis of MPP was established based on the positive of MP- IgM and MP- DNA, complemented by chest radiography on PICU admission. For identifying co-infections, Procalcitonin (PCT) and Lipopolysaccharide (LPS) assays were detected, while blood cultures were used to specify bacterial species; viral co-infections were determined using nucleic acid tests and antibody assays.

The assessed laboratory parameters were white blood cell counts, C-Reactive Protein (CRP), ferritin, Activated Partial Thromboplastin Time (APTT), Fibrinogen (Fib), Alanine Aminotransferase (ALT), Total Bilirubin (TBIL), Lactate Dehydrogenase (LDH), and γ-Glutamyl Transpeptidase (γ-GT).

### Blood sampling

Blood samples were collected in sterile vacutainer tubes on PICU admission, and the serum was subsequently obtained and stored at -80°C.

### Serum S100A8/A9 analysis

Serum S100A8/A9 levels were measured with a specific ELISA kit ((ELH-S100A8-9 RayBiotech, Peachtree Corners, Georgia, USA)). To ensure accurate quantification within the dynamic range of the assay (35–8,000 pg/mL), all serum samples were pre-diluted 8,000-fold based on preliminary tests. The procedure followed the manufacturer's protocol, involving incubation in antibody-pre-coated wells. The measured values were converted to the final serum concentrations (reported in µg/mL) by applying the 8,000-fold dilution factor. The ELISA kit exhibited high precision, with intra-assay Coefficients of Variation (CVs) ≤ 10% and inter-assay CVs ≤ 12% as verified by repeated measurements of three quality control standards.

### Statistical analyses

Data processing was conducted using Stata/SE, version 15.1 (StataCorp LLC). Continuous variables that followed a normal distribution were presented as mean ± Standard Deviation (SD), and inter-group comparisons were made using the student *t*-test. Non-normally distributed data were expressed as the medians and the interquartile range ([IQR]; 25^th^ and 75^th^ data percentiles), with the Wilcoxon Ranksum test applied for between-group comparisons. Categorical data were reported as counts or percentages. Using multivariate logistic regression analysis, the independent risk factors of SMPP were evaluated by using the variables with statistical significance identified in the univariate analysis. Additionally, the Receiver Operating Characteristic (ROC) curve was utilized to analyze the value of independent risk factors in predicting SMPP. A p-value of less than 0.05 was considered statistically significant.

## Results

### Basic characteristics

**Fig. 1:**
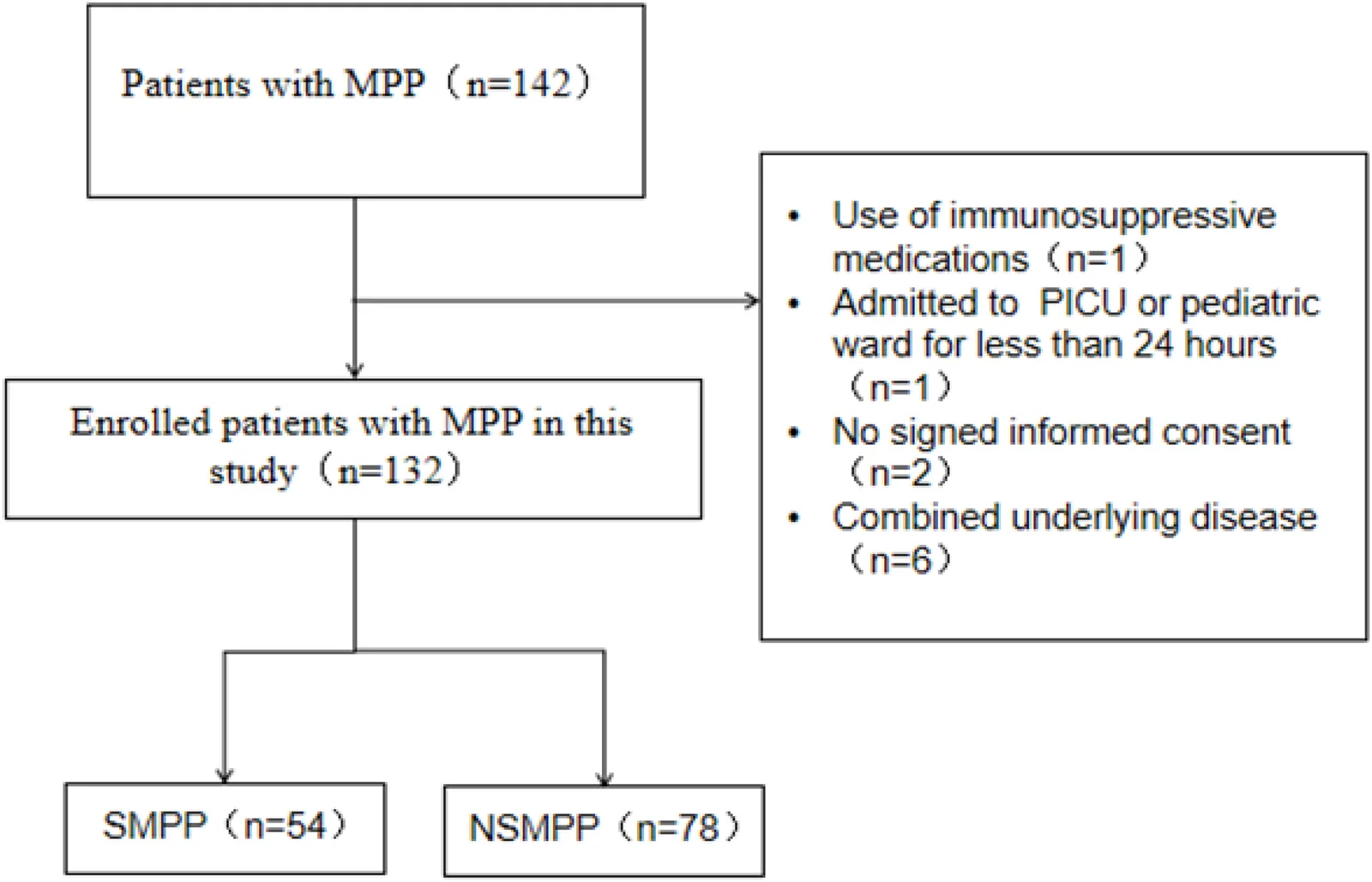
Flowchart of patient enrollment in this study.

During a 6-month period, 142 patients diagnosed with MPP were assessed for eligibility. Exclusions were made for one patient who used immunosuppressive drug, one admitted to PICU for less than 24-hours, two patients who did not sign informed consent, and six cases with underlying diseases. Consequently, 132 pediatric patients with MPP were included in this study, and 54 patients were diagnosed with SMPP and 78 with NSMPP (Fig. 1). The cohort consisted of 71 males and 61 females, ranging in age from 1 to 14 years, with a median age of 7-years (interquartile range: 6‒8.8 years). Among them, 65.9% were aged 5‒9 years, 17.4% were under 5-years-old, and 16.7% were aged 10- to 18-years.

### Comparison of clinical features of SMPP and NSMPP

No significant differences were observed in age and the proportion of co-infections between the SMPP and NSMPP groups (Table 1). The duration of fever and hospital stay in the SMPP group were significantly longer than in the NSMPP group (*p* < 0.001 and *p* = 0.014, respectively). Additionally, the incidence of MP-associated organ injuries, including hypoxemia, pleural effusion, and lung necrosis, was higher in the SMPP group compared to the NSMPP group (Table 1). Notably, the proportion of macrolide-unresponsive cases in the SMPP group was 87%, which was significantly higher than the 69.2% observed in the NSMPP group (*p* = 0.018) (Table 1).

**Table 1:** Univariate analysis of clinical characteristic between SMPP and NSMPP.

Variable	SMPP (*n* = 54)	NSMPP (*n* = 78)	p-value
**Demographic information**			
Male, n (%)	30 (55.6)	41(52.6)	0.736
Age, month, median (Q1-Q3)	84 (72, 108)	84 (72, 106)	0.591
**Clinical features**			
Length of fever, day, median (Q1-Q3)	8 (7, 10)	5 (3, 6)	<0.001
Length of hospitalization, day, median (Q1-Q3)	7 (6, 8)	6 (5, 7)	0.014
**Associated organ damage, n (%)**			
Hypoxemia	7 (12.9)	0 (0)	0.001
Atelectasis	0 (0)	1 (1.2)	0.405
Pleural effusion	15 (27.7)	9 (11.5)	0.018
Pulmonary necrosis	3 (5.5)	0 (0)	0.036
Skin manifestations	1 (1.8)	2 (2.5)	0.788
Cardiovascular disorder	6 (11.1)	6 (7.6)	0.503
Hematologic disorder	2 (3.7)	2 (2.5)	0.708
**Coinfection, n (%)**			
Bacterial	3 (5.6)	9 (11.5)	0.242
Viral	11 (20.3)	16 (20.5)	0.984
Bacterial and viral coinfection	3 (5.5)	4 (5.1)	0.915
**Macrolide -unresponsive MP, n (%)**	47 (87.0)	54 (69.2)	0.018

### Comparison of serum S100A8/A9 levels and other laboratory index in patients of SMPP and NSMPP group at the time of admission

Upon hospital admission, the levels of WBC, neutrophils, CRP, myoglobin, CK-MB, LDH, ALB and IgM in the SMPP group were significantly higher than those in the NSMPP group (all *p* < 0.05). Additionally, PT, APTT, D-dimer, and other indices in SMPP children exceeded those in NSMPP children (all *p* < 0.05). The serum S100A8/A9 levels were elevated in the SMPP group compared to the NSMPP group (5. 877 [3.202, 9.257] vs. 4.349 [2.495, 6.902] μg/mL; *p* = 0.049) (Table 2).

**Table 2:** The laboratory parameters of patients with MPP.

Variable	SMPP (*n* = 54)	NSMPP (*n* = 78)	p-value
**S100A8/A9 (μg/mL)**	5. 877 (3.202, 9.257)	4.349 (2.495, 6.902)	**0.049**
**Blood routine test**			
WBC (× 10^9^/L)	8.125 (5.770, 11.170)	6.860 (5.700, 8.440)	**0.043**
Neutrophils (× 10^9^/L)	5.215 (3.320, 8.840)	3.855 (2.760, 5.870)	**0.008**
CRP (mg/L)	10.5 (5.0, 16.0)	6.5 (5.0, 12.0)	**0.009**
HB (g/L)	124.148±8.930	123.821±8.394	0.830
PLT(× 10^9^/L)	349 (278, 403)	319 (255, 384)	0.169
**Blood biochemical test**			
ALT (U/L)	13.5 (11.0, 19.0)	14 (12.0, 18.0)	0.577
AST (U/L)	29.5 (25.0, 33.0)	29 (24.0, 34.0)	0.753
CK (U/L)	84.0 (45.0, 149.0)	68.5 (41.0, 93.0)	0.052
CK- MB (U/L)	19 (15, 23)	22 (17, 28)	**0.017**
LDH (U/L)	329.5 (288.0, 365.0)	302 (265.0, 356.0)	**0.036**
Myoglobin (ng/mL)	10.21 (7.72, 13.57)	8.76 (6.99, 10.42)	**0.017**
BNP (pg/mL)	41.55 (7.40, 78.90)	26.9 (9.20, 93.90)	0.915
Troponin (ng/mL)	0.02 (0.02, 0.05)	0.02 (0.02, 0.06)	0.433
ALB (g/L)	40.67 (39.02, 42.35)	42.15 (40.73, 43.56)	**0.002**
Ferritin (ng/mL)	178.0 (127.0, 244.0)	152.0 (119.0, 173.5)	0.053
PT (s)	12.15 (11.40, 12.70)	11.80 (11.30, 12.30)	**0.043**
APTT (s)	29.65 (28.20, 31.90)	28.80 (27.40, 31.50)	**0.035**
Fib (g/L)	4.50 (3.89, 5.52)	4.06 (3.46, 5.30)	0.144
PCT (ng/mL)	0.09 (0.05, 0.15)	0.08 (0.05, 0.13)	0.058
D-dimer (mg/L)	0.57 (0.31, 0.93)	0.37 (0.23, 0.48)	**0.001**
**Cytokine**			
IL‐6 (pg/mL)	5.22 (2.96, 16.76)	4.27 (3.17, 7.60)	0.130
IL-8 (pg/mL)	24.23 (13.23, 40.18)	24.45 (12.42, 42.67)	0.924
IFN-γ (pg/mL)	38.08 (19.13, 62.06)	33.90 (21.27, 59.20)	0.723
IFN-α (pg/mL)	6.90 (4.63, 12.85)	7.66 (3.50, 10.77)	0.374
TNF-α (pg/mL)	6.87 (3.96, 13.09)	6.05 (3.17, 10.77)	0.407
**Cellular immunity**			
CD3^+^ (%)	66.80 (60.07, 70.95)	67.24 (59.85, 71.44)	0.722
CD4^+^ (%)	35.561±8.129	35.131±7.999	0.692
CD8^+^ (%)	24.179±4.532	24.850±4.688	0.417
**Immunoglobulin**			
IgG (g/L)	11.196±2.904	11.202±2.516	0.989
IgA (g/L)	1.656±0.669	1.533±0.562	0.872
IgM (g/L)	1.64 (1.30, 2.08)	1.37 (1.07, 1.77)	**0.022**

WBC, White Blood Cell; CRP, C-Reactive Protein; HB, Hemoglobin; PLT, Platelets; ALT, Glutamic-pyruvic Transaminase; AST, Aspartate Amino Transferase; CK, Creatine Kinase; CK-MB, Creatine kinase-MB; LDH, Lactate Dehydrogenase; BNP, Brain Natriuretic Peptide; ALB, Albumin; APTT, Activated Partial Thromboplastin Time; Fib, Fibrinogen; TNF, Tumor Necrosis Factor; IL, Interleukin; PCT, Procalcitonin.

### Multivariate logistic regression analysis on risk factors of SMPP

The multivariate *logistic* regression analysis was conducted on the serum S100A8/A9 level, CRP, D-dimer, and APTT. The results revealed that a high level of S100A8/A9 (*p* = 0.049) is an independent risk factor for SMPP (Table 3).

**Table 3:** Multivariate logistic regression analysis of the risk factors of SMPP.

Variable	β	SE	Waldχ^2^	OR (95% CI)	p-value
S100A8/A9	0.793	0.040	3.860	1.083 (1.000‒1.172)	0.049
CRP	0.288	0.016	3.170	1.029 (0.997‒1.062)	0.075
D-dimer	-0.003	0.074	0.010	0.997 (0.862‒1.153)	0.968
APTT	0.104	0.056	0.063	1.109 (0.994‒1.238)	0.063

### Predictive value of MPP-related detection index for SMPP

**Fig. 2:**
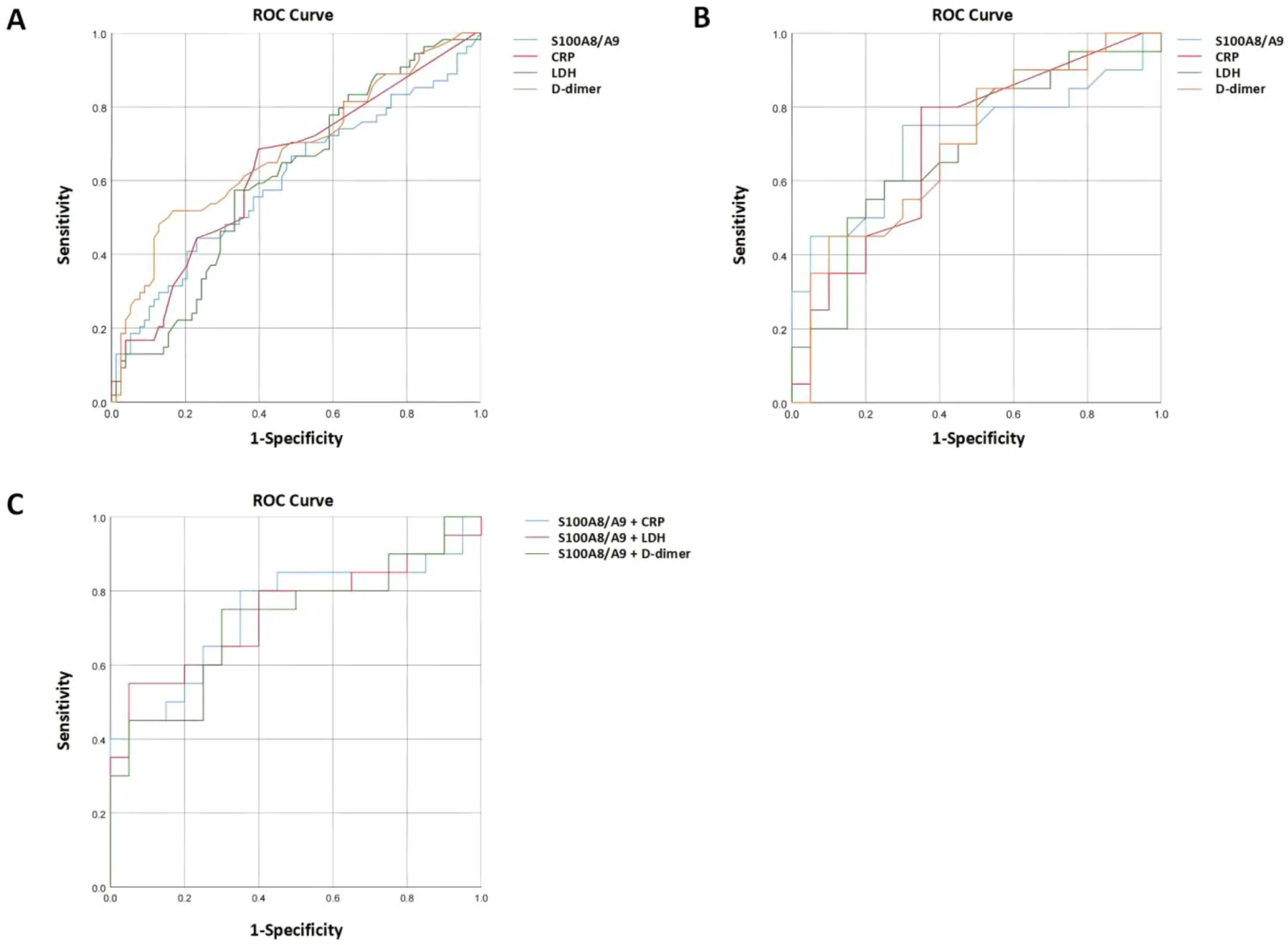
(A) ROC curves of S100A8/A9, CRP, LDH, D-dimer for predicting SMPP. (B‒C) The ROC curve for predicting SMPP was based on 40 cases of age, sex, and co-existing bacterial or viral infection. (B) Single predictors including S100A8/A9, CRP, LDH and D-dimer; (C) S100A8/A9+CRP, S100A8/A9+LDH, S100A8/A9+ D-dimer joint index.

Subsequently, Receiver Operating Characteristic (ROC) analysis was employed to assess the diagnostic utility of serum S100A8/A9 levels in predicting SMPP. The ROC curve established cut-off values for S100A8/A9, CRP, LDH, and D-dimer at 7.165 μg/mL, 7.000 mg/L, 323.000 U/L, and 0.570 mg/L respectively. As shown in Table 4 and Fig. 2A, S100A8/A9 does not exhibit the highest predictive value, with a sensitivity of 44.4% and specificity of 76.9%, compared to the other three markers. Consequently, it is considered that children with MPP often experience co-infections of bacterial and viral origins.

**Table 4:** Predictive value of S100A8/A9, CRP, LDH, and D-dimer in children with SMPP.

**Variables**	**Cut-off value**	**AUC**	**Sensitivity (%)**	**Specificity (%)**	**95% CI**	**p-value**
S100A8/A9	7.165	0.601	44.4	76.9	0.512‒0.685	0.052
CRP	7.000	0.631	68.5	60.3	0.543‒0.714	0.007
LDH	323.000	0.608	57.4	66.7	0.519‒0.691	0.030
D-dimer	0.570	0.678	48.1	87.2	0.591‒0.756	<0.001

### Serum S100A8/A9 is an additional predictor of SMPP after adjusted by age, sex, and co-infections

To mitigate potential confounders such as age, sex, and co-existing bacterial and viral infections, 20-pairs of cases were matched from a pool of 132-cases using the ccmatch algorithm. To assess the diagnostic potential of S100A8/A9, CRP, LDH, and D-dimer in children with SMPP, ROC analysis was conducted. As shown in Fig. 2B and Fig. 2C, the AUC for S100A8/A9 was 0.714 (95% CI: 0.549‒0.845), which is comparable to the predictive utility of CRP (AUC = 0.701 [95% CI: 0.536‒0.835]), LDH (AUC = 0.690 [95% CI: 0.524‒0.826]), and D-dimer (AUC = 0.698 [95% CI: 0.532‒0.832]).For the assessment of SMPP, the specificity and sensitivity were 70% and 75%, respectively, at a cutoff value of 4.067 μg/mL (Table 5 and Fig. 2B). The AUC matched by the 4 factors is higher than the original AUC (0.601 [95% CI: 0.512‒0.685]) (Table 4 and Fig. 2A). Decision curve analysis suggests that the predictive utility of S100A8/A9 surpasses the other three factors. Considering the associations of serum S100A8/A9, CRP, LDH, and D-dimer with pediatric MPP, the predictive effectiveness of S100A8/A9 alone, and in combination with CRP, LDH or D-dimer was examined and compared. The AUC for S100A8/A9 combined with CRP was 0.742 (95% CI: 0.580‒0.867), suggesting a potential benefit over S100A8/A9 alone (0.714 [95% CI: 0.549-0.845)]), S100A8/A9+LDH (0.738 [95% CI: 0.575-0.864]), and S100A8/A9+D-dimer (0.720 [95% CI: 0.556‒0.850]) (Table 5 and Fig. 2C).

**Table 5:** Predictive value of independent correlation factors in 40 cases matched by age, sex, and comorbid bacteria or viruses.

**Variables**	**Cut-off value**	**AUC**	**95% CI**	**Sensitivity (%)**	**Specificity (%)**	**p-value**
S100A8/A9	4.067	0.714	0.549‒0.845	75	70	0.012
CRP	7.000	0.701	0.536‒0.835	80	65	0.016
LDH	303.000	0.690	0.524‒0.826	60	75	0.027
D-dimer	0.295	0.698	0.532‒0.832	85	50	0.020
S100A8/A9+CRP	0.395	0.742	0.580‒0.867	80	65	0.003
S100A8/A9+LDH	0.565	0.738	0.575‒0.864	55	95	0.004
S100A8/A9+D-dimer	0.426	0.720	0.556-0.850	75	70	0.009

### Comparison of complications between MPP with higher S100A8/A9 group and lower S100A8/A9 group

Based on the results, S100A8/A9 demonstrated the highest predictive value for SMPP. The average level of S100A8/A9 was found to be 4.067 μg/mL, allowing us to categorize patients into two groups: those with elevated S100A8/A9 levels (> 4.067 μg/mL) and a control group with levels ≤ 4.067 μg/mL. When comparing complications between these groups, it was observed that the incidence of fever days and macrolide-unresponsive MP was significantly higher in the elevated S100A8/A9 group compared to the control group. Conversely, no significant differences were found in the incidence of pleural effusion, hypoxemia, dysfunction in the digestive system and circulatory system between the two groups (Table 6). Additionally, the case number of needing flexible bronchoscscopy was significantly more in group of elevated S100A8/A9 levels (> 4.067 μg/mL) (*n* = 4) than that in a control group with levels ≤ 4.067 μg/mL (*n* = 0) (Table 6).

**Table 6:** Complications comparison between elevated S100A8/A9 group and control group.

**Complications**	**S100A8/A9 >4.067 μg/mL (***n* = **21)**	**S100A8/A9 ≤4.067 μg/mL (***n* = **19)**	**p-value**
Pleural effusion, n (%)	7 (33.3)	3 (15.7)	0.206
Hypoxemia, n (%)	1 (4.7)	0 (0)	0.342
Gastrointestinal system dysfunction, n (%)	1 (4.7)	0 (0)	0.342
Cardiovascular system dysfunction, n (%)	1 (4.7)	1 (5.2)	0.943
Fever days, median (Q1-Q3)	7 (7, 9)	5 (2, 7)	**0.005**
Macrolide-unresponsive MP, n (%)	21 (100)	11 (57.8)	**0.001**
Azithromycin, n (%)	16 (76.1)	12 (63.1)	0.375
Tetracycline, n (%)	6 (28.5)	9 (47.3)	0.226
Quinolone, n (%)	16 (76.1)	9 (47.3)	0.063
Systemic steroids, n (%)	19 (90.4)	17 (89.4)	0.917
Oxygen support, n (%)	2(9.5)	0	0.173
IVIG, n (%)	1(4.7)	0	0.342
Flexible bronchoscscopy, n (%)	4(19.0)	0	**0.048**

IVIG, Immunoglobulin.

## Discussion

SMPP are more susceptible to serious pulmonary and extrapulmonary complication.^17^ Previous research indicated that delays in receiving appropriate treatment correlated with the progression of more severe conditions.^18^^,^^19^ Critically ill children exhibit more pronounced inflammatory response and extrapulmonary complications, coupled with decreased adaptive immunity.^20^ The presently study found that serum S100 A8/A9 levels related with the severity of MPP in children. Furthermore, the combination of S100A8/A9 with CRP provides more value in assessing the severity of MPP compared to a single routine biomarker.

MPP mainly occurs in school-age population. Our previous study reported that 77.3% of SMPP cases in the PICU involved children younger than 6-years.^21^ In this study, 33.3% of the SMPP cases were in children under 6-years-old. Moreover, among 163 patients tested for macrolide resistance, 90.2% (147/163) were infected with macrolide-resistance MP.^21^ In this study, a 76.5% (101/132) infection rate with macrolide-resistance strains indicates the main issue about drug-resistant *Mycoplasma pneumoniae* infections.

Due to the complications associated with SMPP, it is imperative to identify SMPP to prevent worsening outcomes. Cytokines might serve as potential markers of SMPP and early intervention in immune response regulation could mitigate SMPP.^22^ Additionally, increased D-dimer (AUC = 0.721 [95% CI: 0.572‒0.786]) and serum CXCL10/IP-10 levels positively correlated with the severity of MPP.^23^^,^^24^ And serum D-dimer levels greater than > 3.705 mg/L is an independent predictor of MPP combined with necrotizing pneumonia.^25^ In the present study, before matching, the AUC for D-dimer for predicting SMPP was 0.678 (95% CI: 0.591‒0.756) with a sensitivity of 48.1% and a specificity of 87.2%. After matching, the AUC for D-dimer for predicting SMPP was 0.698 (95% CI: 0.532‒0.832) with a sensitivity of 85% and a specificity of 50%. Our research results are largely consistent with previous studies (D-dimer, AUC = 0.721 [95% CI: 0.572‒0.786]).^23^ However, the limitations of above indicators lie in their difficulty in distinguishing MPP from non-MPP. (e.g., bacteria, virus). The shortage of early warning indicators for SMPP persists, and neither the sensitivity nor the specificity is high.

S100A8/A9 have been identified as potential biomarkers for inflammatory diseases such as juvenile arthritis, Inflammatory Bowel Disease (IBD), and autoimmune disorders.^13^^,^^15^^,^^26^ Moreover, S100A8/A9 also show promise in sepsis-related organ injury,^27^ cancer prognosis,^28^ and severity stratification in COVID-19.^29^ As conserved Damage-Associated Molecular Pattern (DAMP) proteins, S100A8/A9 exert their biological effects primarily through interactions with Toll-Like Receptor-4 (TLR4) and the receptor for Receptor of Advanced Glycation Endproducts (RAGE). These interactions drive the recruitment and activation of innate immune cells, thereby amplifying inflammatory responses.^26^ In the setting of MPP, elevated levels of S100A8/A9 are closely linked to the severity of localized pulmonary inflammation and associated immune dysregulation.^30^ Existing studies have shown that S100A8/A9 exhibits higher tissue specificity and clinical diagnostic value in MPP, particularly in distinguishing the severity of *pneumonia.*^31^ In MPP, elevated S100A8/A9 in Bronchoalveolar Lavage Fluid (BALF) suggests diagnostic and pathogenic roles, potentially through promoting epithelial apoptosis.^15^ However, the association between S100A8/A9 and SMPP, as well as the impact of co-infection with bacteria, viruses, and other factors, remains unexplored. In our study, serum S100A8/A9 levels were significantly higher in SMPP than in NSMPP (*p* < 0.05). Though the AUC for S100A8/A9 is 0.601 (95% CI: 0.512‒0.685) with a sensitivity of 44.4% and a specificity of 76.9% before matching, the AUC was up to 0.714 (95% CI: 0.549‒0.845) with a sensitivity of 75% and a specificity of 70% after matching. These findings indicate that S100A8/A9 possesses greater sensitivity and specificity than D-dimer for predicting SMPP. The observed variations in AUC and optimal cut-off values may be influenced by the statistical adjustments for cofactors, particularly co-infections. Therefore, S100A8/A9 appears to have a more promising independent predictive value for SMPP compared to D-dimer when bacterial or viral co-infection is not a confounding factor. Moreover, patients with serum S100A8/A9 levels greater than 4.067 μg/mL had a longer duration of fever, higher macrolide-unresponsive, and a higher proportion of patients requiring flexible bronchoscopy. It is the first report about the crucial role of serum S100A8/A9 as an additional predictor for SMPP, possibly associated with macrolide unresponsive.

While prior studies^32^^,^^33^ and our data confirm associations between CRP/D-dimer and SMPP, their relatively low specificities (65% and 50%, respectively) limit their utility in early differentiation and risk stratification. In contrast, S100A8/A9 shows higher specificity (70%) for SMPP, suggesting its potential as a more SMPP-specific biomarker. Methodologically, immunoturbidimetric measurement of CRP and D-dimer requires 2 mL of blood, whereas S100A8/A9 detection by ELISA uses only 2 μL of serum. This minimal serum volume makes S100A8/A9 testing especially suitable for pediatric patients with limited blood volume and supports the development of future point-of-care testing. Cost analysis shows that its per-test expense is comparable to conventional methods (30‒50 RMB), supporting its translational potential. We hypothesize that S100A8/A9 delivers complementary information to established biomarkers. A multi-marker panel comprising CRP, D-dimer, and S100A8/A9 would simultaneously reflect systemic inflammation, coagulation activation, and specific pulmonary damage, potentially improving the sensitivity and specificity of SMPP risk prediction and prognostic assessment. Suah an approach could enhance early SMPP detection, guide individualized intervention, and inform precision care strategies in children.

### Limitation

There were several limitations in this study. First, it must be emphasized that our findings are derived from a limited single-center matched cohort and should be regarded as exploratory, and further validation through larger-scale, prospective, multicenter studies is necessary to establish generalizability. Second, the inclusion criteria were restricted to hospitalized patients, which inevitably excluded those with milder MPP who did not require hospitalization, thereby limited the generalizability of the findings to a broader population. Third, although blood samples were collected and serum S100A8/A9 levels were measured at admission, dynamic monitoring during hospitalization was not performed. Consequently, it was not possible to elucidate the change patterns of this biomarker throughout the disease course or its response to treatment, which constrains a deeper understanding of its dynamic role in disease pathophysiology. Forth, this matched-cohort study controlled for key variables such as age, sex, and co-infection status, potential confounding factors such as including prior antibiotic use, vaccination status, and nutritional status were not assessed. These unmeasured variables may influence baseline levels of inflammatory markers such as S100A8/A9 and CRP, thereby affecting the evaluation of their independent predictive value. Given the small size of the matched cohort, the findings are of an exploratory nature and require further validation in larger, multicenter prospective studies. Future studies should aim to design prospective study that include patients with varying disease severity and incorporate serial biological sample collection to comprehensively elucidate the natural history, risk factors of MPP, and the dynamic role of S100A8/A9 therein.

## Conclusion

S100A8/A9 serves as an additional predictor for assessing SMPP in pediatric patients, which appears to have a higher sensitivity and specificity for SMPP prediction compared to that for D-dimer when bacterial or viral co-infection is not a confounding factor. Future prospective studies should incorporate serial measurements of S100A8/A9 to elucidate its kinetics and evaluate its potential for monitoring treatment response and predicting disease progression.

## Ethics approval and consent to participate

The study protocol received approval from the Ethics Committee of Children's Hospital affiliated to Shanghai Jiao Tong University (approval n° 2023R099-F01). Informed consent was obtained from the patients' parents or relatives.

## Authors’ contributions

Zihui Liu: Data curation; validation; writing-original draft preparation. Lihui Gao: Data curation; visualization; writing-original draft preparation. Yun Cui: Writing-review & editing; investigation. Yiping Zhou: Writing-review & editing; supervision. Xi Xiong: Software; validation. Chunxia Wang: Formal analysis; writing-reviewing and editing. Yucai Zhang: Conceptualization; methodology.

## Funding

This study was supported by the Shanghai Municipal Health Commission Special Project for Clinical Research in the Healthcare Industry (20244Y0020), the National Natural Science Foundation of China (82100085), the National Key Research and Development Program of China (2021YFC2701800, 2021YFC2701805, 2021YFC2701704, 2021YFC2701700), the Science and Technology Commission of Shanghai Municipality (18411951000 and 21Y11902600), the Shanghai Municipal Commission of Health and Family Planning (202340076), the Seed Program for Research and Translation of Medical New Technologies by the ‌Shanghai Municipal Commission of Health and Family Planning (2024ZZ2007) and the Opening Project Fund of National Facility for Translational Medicine (Shanghai) (TMSK-2024-112).

## Data availability statement

The datasets used and/or analyzed during the current study are available from the corresponding author upon reasonable request.

## Declaration of competing interest

The authors declare no conflicts of interest.
